# Supplementary material for: Studying the cellular basis of small bowel enteropathy using high-parameter flow cytometry in mouse models of primary antibody deficiency
Source: Front Immunol. 2024 May 13;15:1278197. doi: 10.3389/fimmu.2024.1278197 (PMC11128607; doi:10.3389/fimmu.2024.1278197)
Supplement: Supplementary file 1 [file DataSheet_1.pdf]

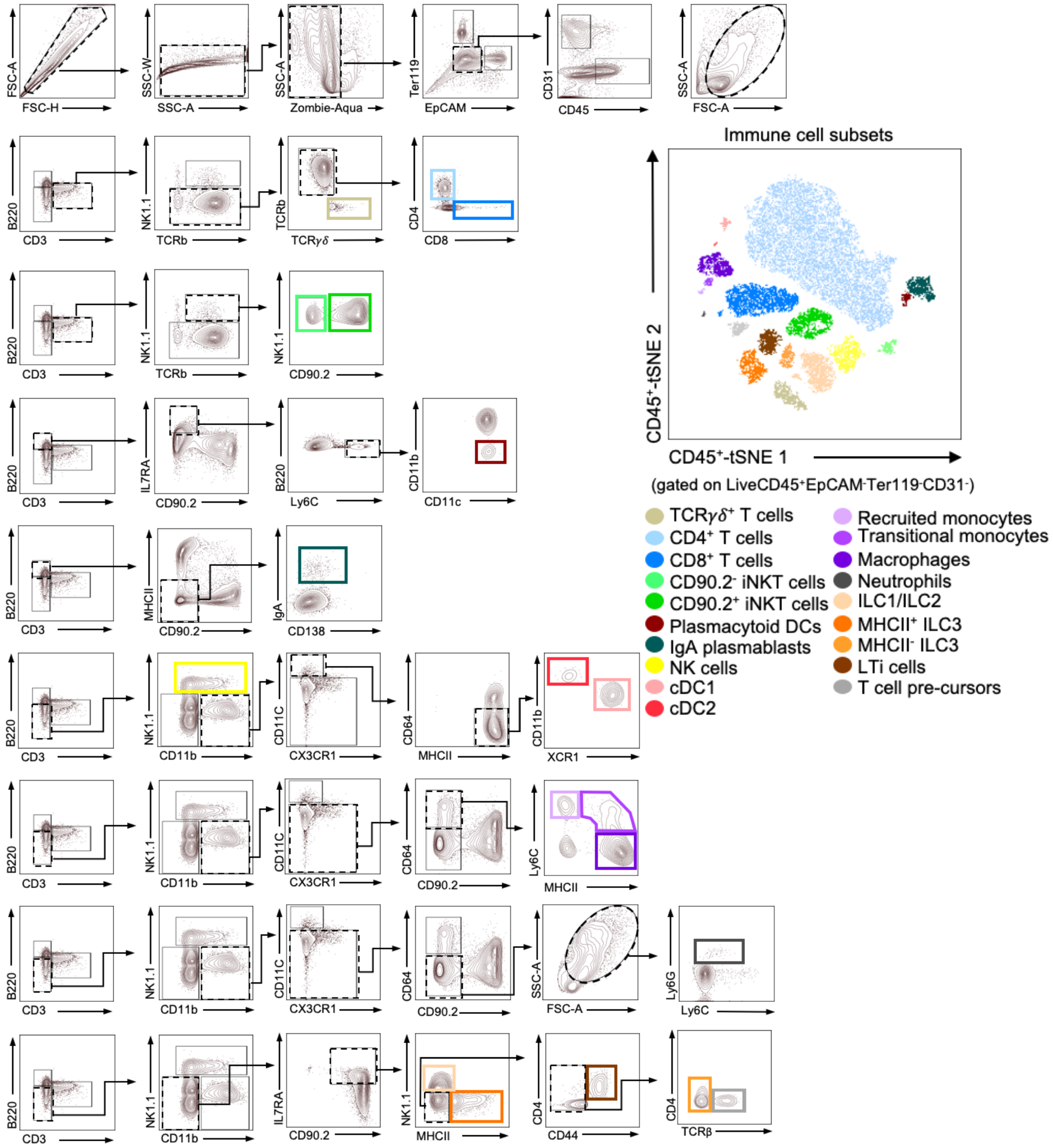

Supplementary Figure S1. Gating rubric for immune cells. Gating rubrics used to calculate absolute abundance of immune cell subsets are shown. Terminal gates defining tSNE cell clusters are color-coded. tSNE plot used in Figure 2A and population names corresponding to terminal gates are provided.

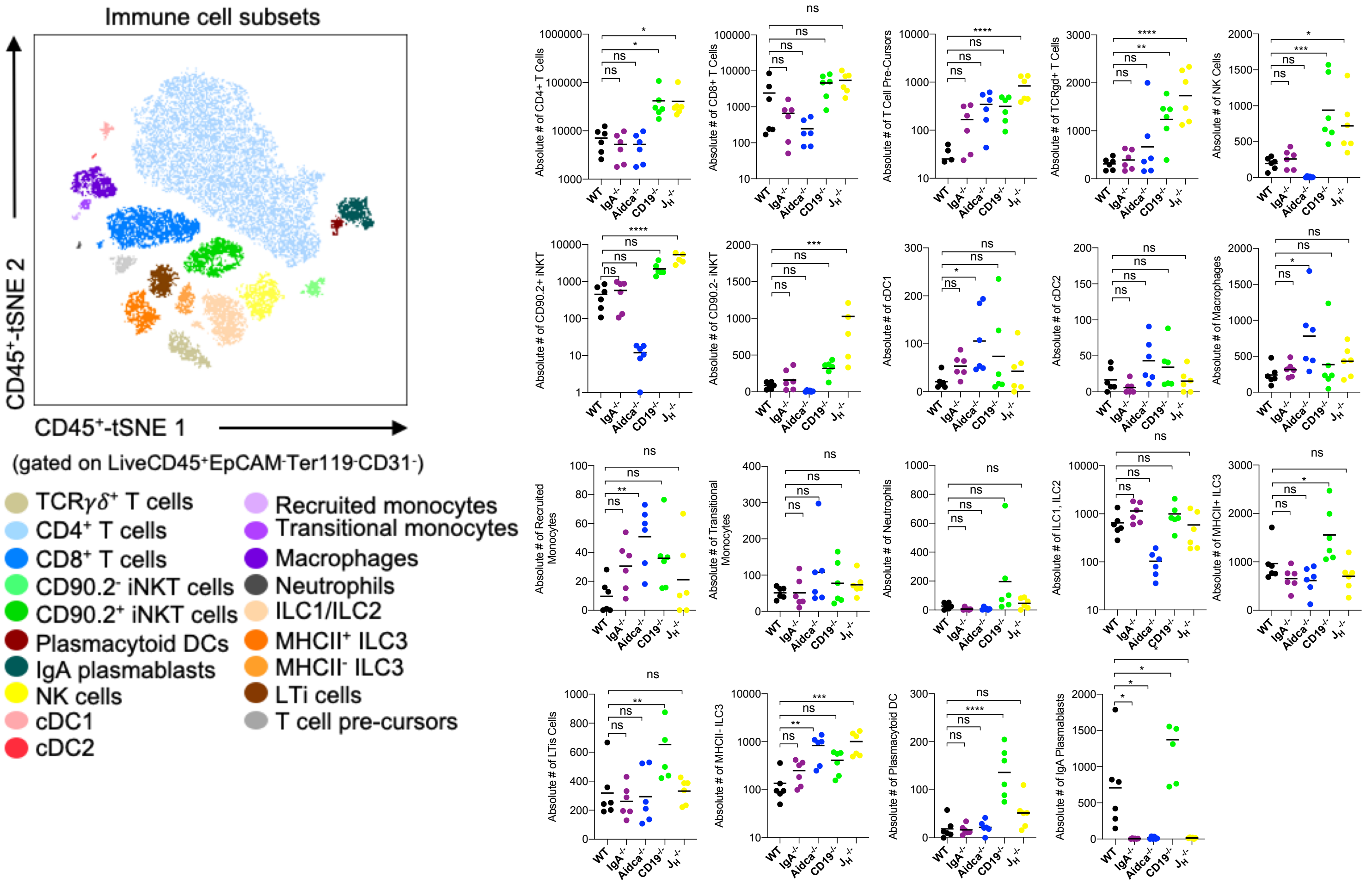

Supplementary Figure S2. Statistical comparisons of all cellular subsets derived from our general immune panel. Dunnett's Test ("all vs. WT"), ns=p>0.05, *=p<0.05, **=p<0.01, ***=p<0.001, ****=p<0.0001.

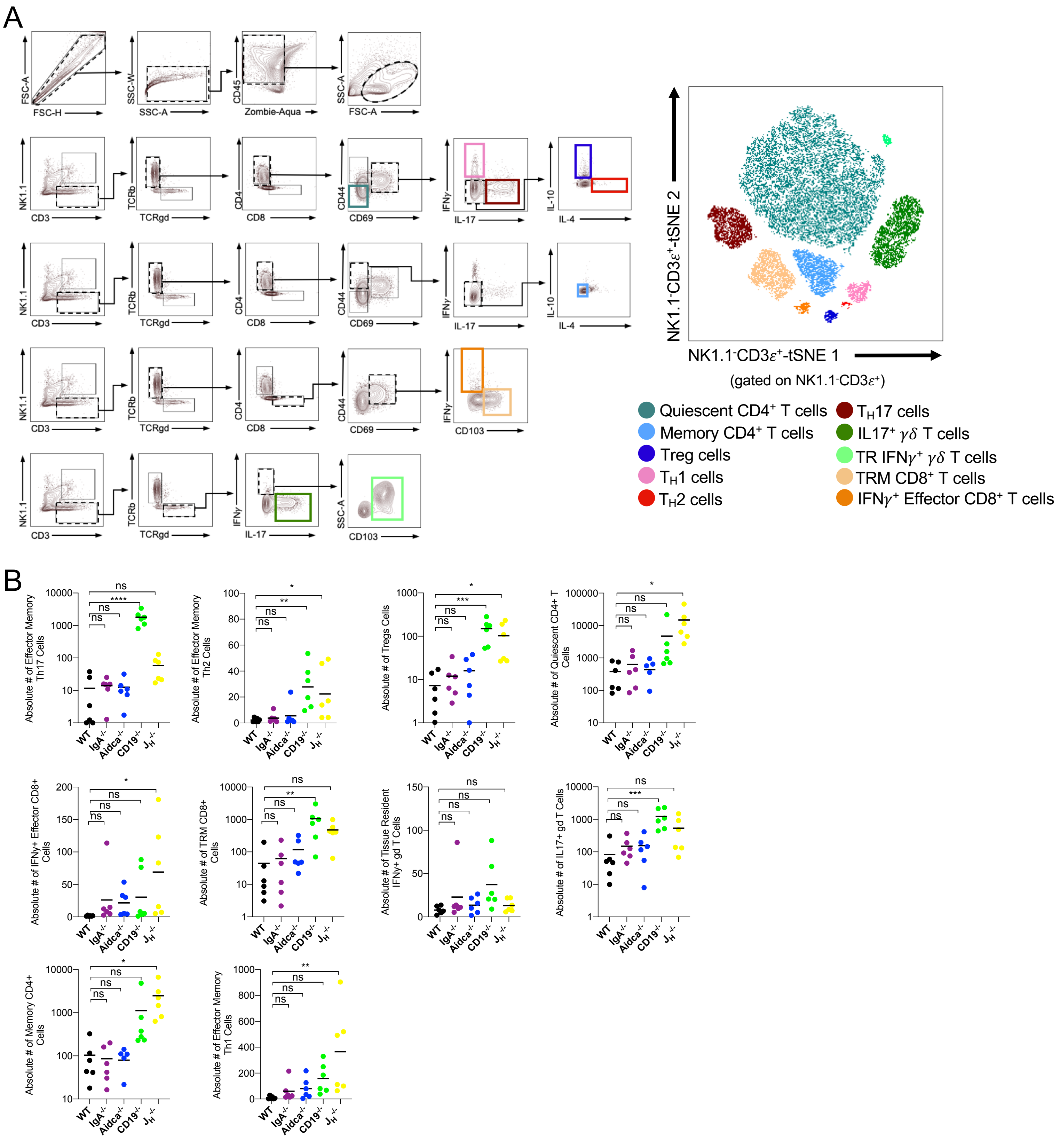

Supplementary Figure S3 Gating rubric and statistical comparisons of conventional T cells. (A) Gating rubrics used to calculate absolute abundance of conventional T cell subsets are shown. Terminal gates defining tSNE cell clusters are color-coded. tSNE plot used in Figure 3A and population names corresponding to terminal gates are provided. **(B)** Statistical comparisons of conventional T cell subsets are provided. Dunnett's Test ("all vs. WT"), ns=p>0.05, *=p<0.05, **=p<0.01, ***=p<0.001, ****=p<0.0001.

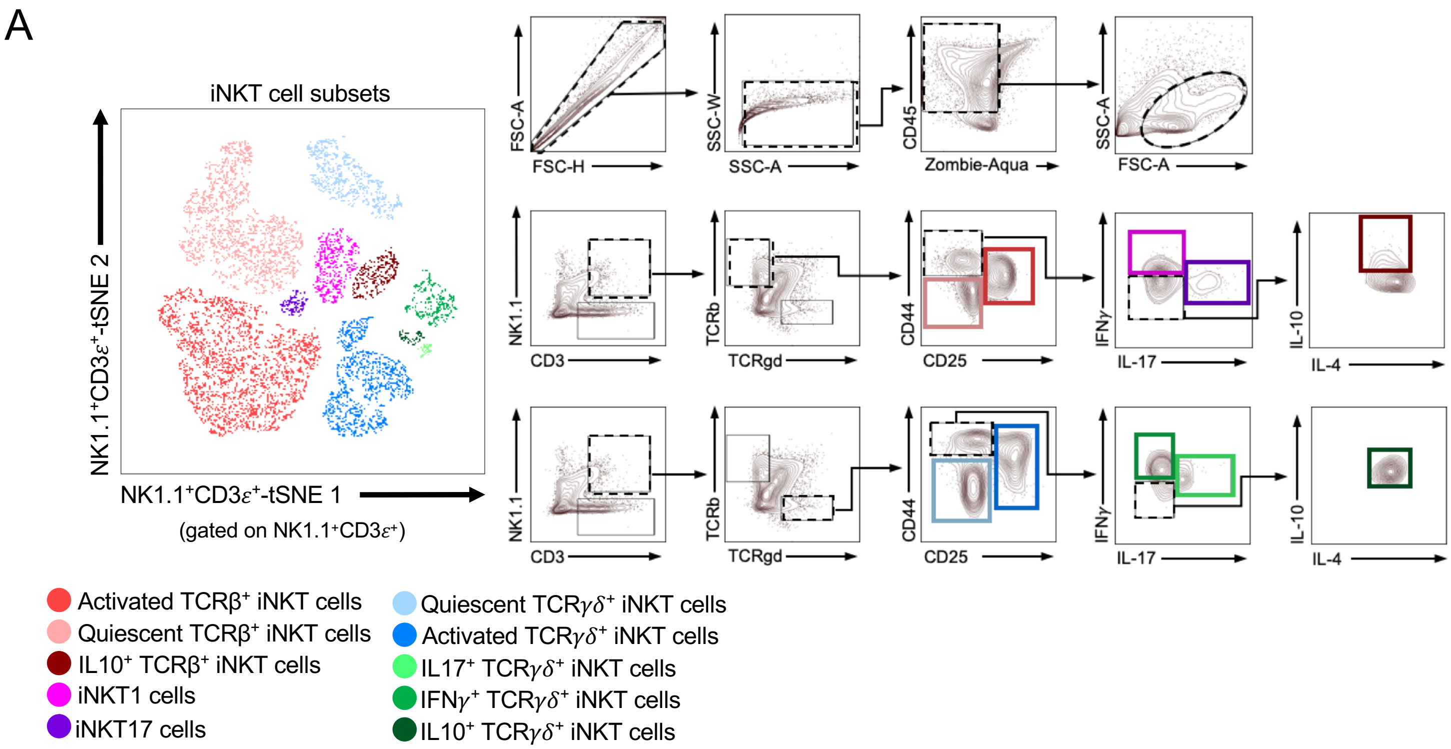

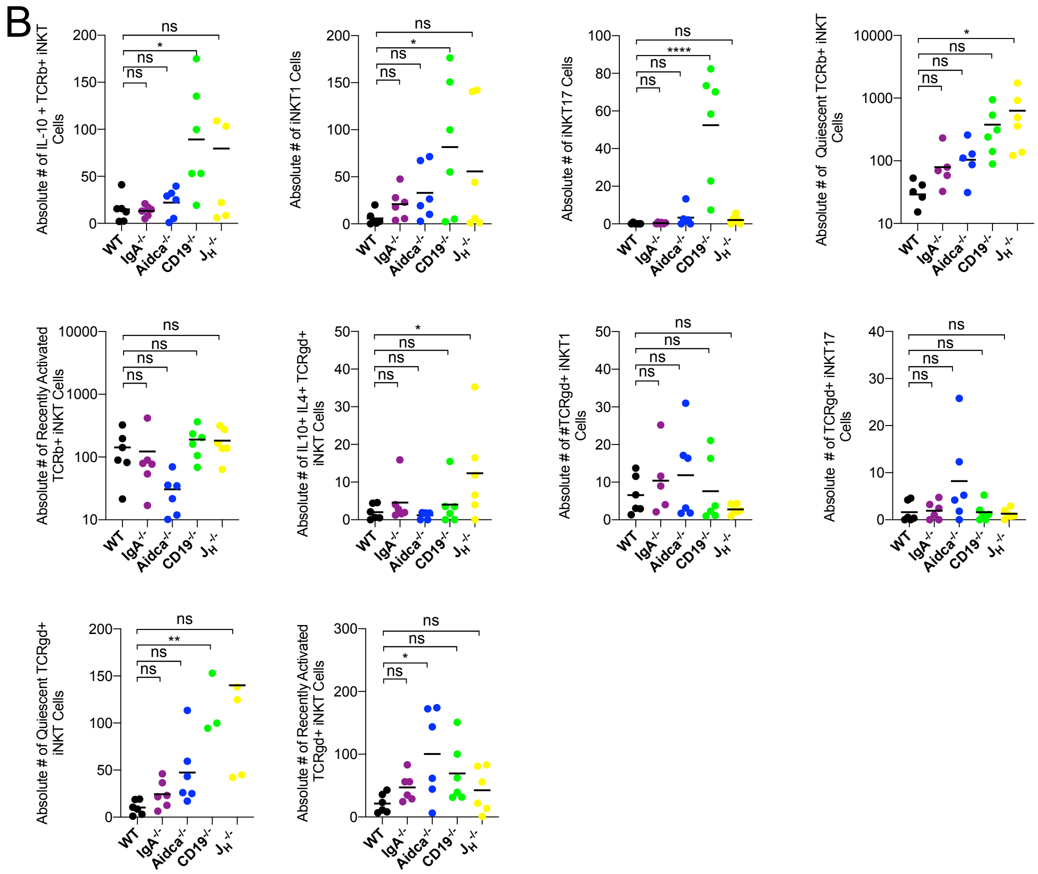

Supplementary Figure S4. Gating rubric and statistical comparisons of invariant NKT cells. (A) Gating rubrics used to calculate absolute abundance of invariant NKT cell subsets are shown. Terminal gates defining tSNE cell clusters are color-coded. tSNE plot used in Figure 3A and population names corresponding to terminal gates are provided. **(B)** Statistical comparisons of invariant NKT cell subsets are provided. Dunnett's Test ("all vs. WT"), ns=p>0.05, *=p<0.05, **=p<0.01, ***=p<0.001, ****=p<0.0001.

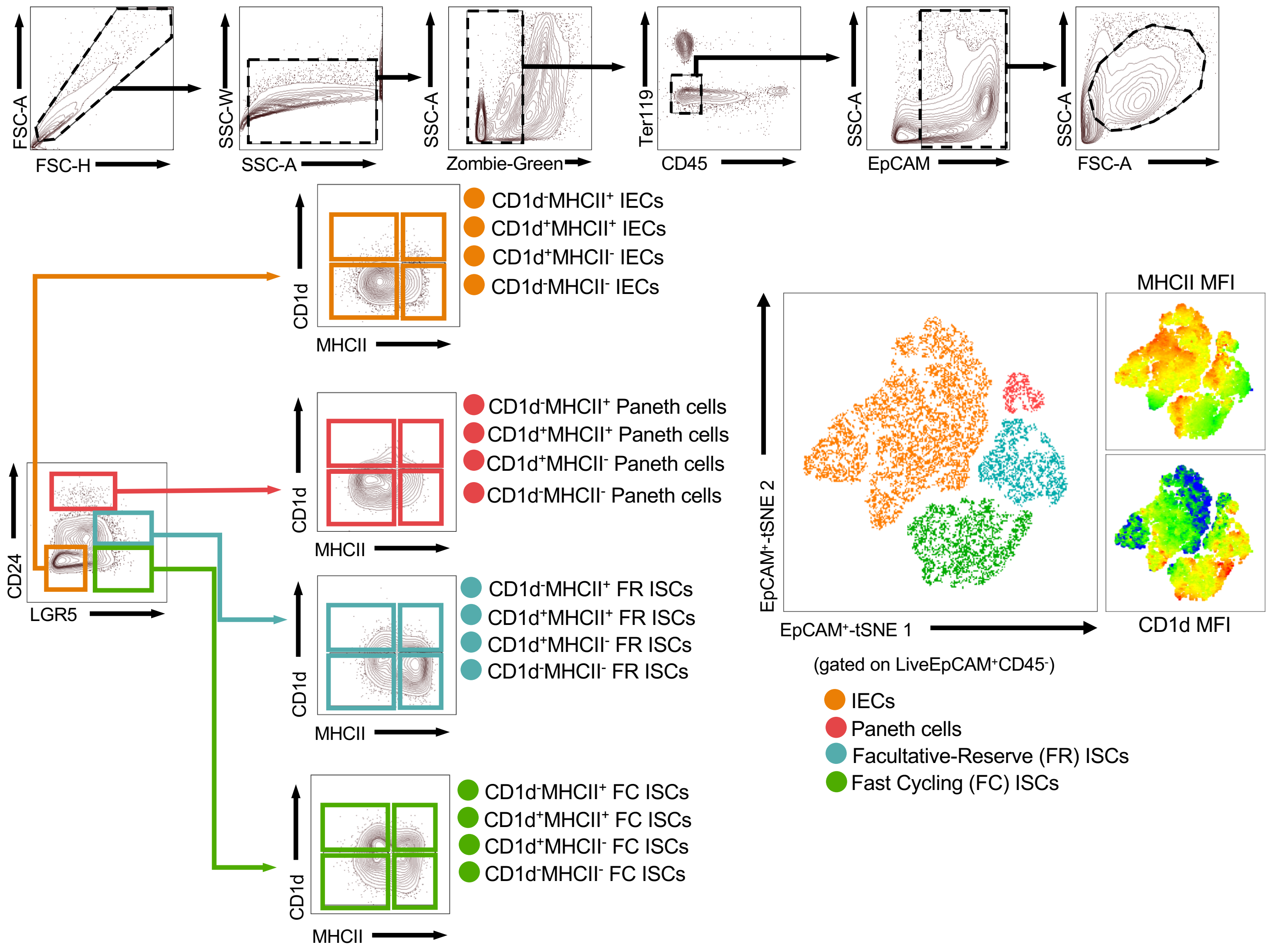

Supplementary Figure S5 Gating rubric for IECs and ISCs. Gating rubrics used to calculate absolute abundance of IECs and ISCs are shown. Terminal gates defining tSNE cell clusters are color-coded. tSNE plot used in Figure 4A and population names corresponding to terminal gates are provided.

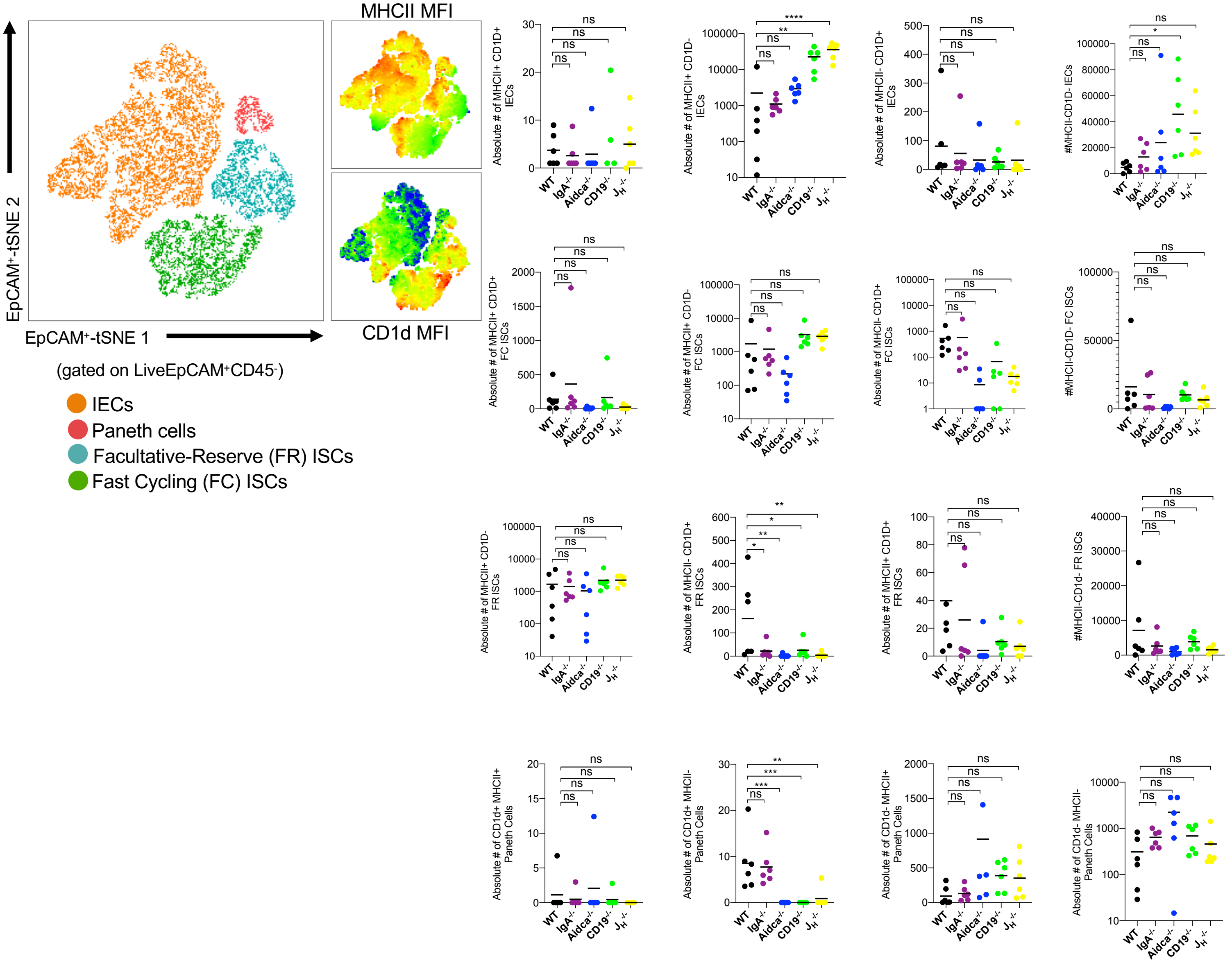

Supplementary Figure S6. Statistical comparisons of all cellular subsets derived from our IEC/ISC panel. Dunnett's Test ("all vs. WT"), ns=p>0.05, *=p<0.05, **=p<0.01, ***=p<0.001, ****=p<0.0001.
